# Gα_12_ Drives Invasion of Oral Squamous Cell Carcinoma through Up-Regulation of Proinflammatory Cytokines

**DOI:** 10.1371/journal.pone.0066133

**Published:** 2013-06-07

**Authors:** Shiou-Ling Jian, Hsin-Yi Hsieh, Chun-Ta Liao, Tzu-Chen Yen, Shu-Wei Nien, Ann-Joy Cheng, Jyh-Lyh Juang

**Affiliations:** 1 Graduate Institute of Life Sciences, National Defense Medical Center, Taipei, Taiwan; 2 Institute of Molecular and Genomic Medicine, National Health Research Institutes, Miaoli, Taiwan; 3 Head and Neck Oncology Group, Chang Gung Memorial Hospital and Chang Gung University, Taoyuan, Taiwan; 4 Graduate School of Medical Biotechnology, Chang Gung University, Taoyuan, Taiwan; 5 Ph.D. Program for Aging, China Medical University, Taichung, Taiwan; Innsbruck Medical University, Austria

## Abstract

Oral squamous cell carcinoma (*OSCC*) ranks among the top ten most prevalent cancers worldwide. Like most head and neck squamous cell carcinomas (HNSCCs), OSCC is highly inflammatory and aggressive. However, the signaling pathways triggering the activation of its inflammatory processes remain elusive. G protein-coupled receptor signaling regulates the inflammatory response and invasiveness of cancers, but it remains unclear whether Gα_12_ is a critical player in the inflammatory cytokine pathway during the tumorigenesis of OSCC. This study was undertaken to determine the role of Gα_12_ signaling in the regulation of proinflammatory cytokines in their mediation of OSCC invasion. We found that both the transcription and protein levels of Gα_12_ are up-regulated in OSCC tumors. The elevated Gα_12_ expressions in OSCC patients also correlated with extra-capsular spread, an indicator of tumor invasiveness in HNSCCs. This clinical finding was supported by the studies of overexpression and RNAi knockdown of Gα_12_ in OSCC cells, which demonstrated that Gα_12_ promoted tumor cell migration and invasion. To understand how Gα_12_ modulates OSCC invasiveness, we analyzed key biological processes in microarray data upon depletion of Gα_12_ and found that cytokine- and other immune-related pathways were severely impaired. Importantly, the mRNA levels of IL-6 and IL-8 proinflammatory cytokines in clinical samples were found to be significantly correlated with the increased Gα_12_ levels, suggesting a potential role of Gα_12_ in modulating the IL-6 and IL-8 expressions. Supporting this hypothesis, overexpression or RNAi knockdown of Gα_12_ in OSCC cell lines both showed that Gα_12_ positively regulated the mRNA and protein levels of IL-6 and IL-8. Finally, we demonstrated that the Gα_12_ promotion of tumor cell invasiveness was suppressed by the neutralization of IL-6 and IL-8 in OSCC cells. Together, these findings suggest that Gα_12_ drives OSCC invasion through the up-regulation of IL-6 and IL-8 cytokines.

## Introduction

Head and neck squamous cell carcinomas (HNSCCs) rank as the sixth most prevalent cancer worldwide, affecting up to 600,000 people each year [1], [2]. Among the various HNSCC subtypes, about 10% are accounted by oral squamous cell carcinoma (*OSCC*) [1]. However, the overall survival rate for OSCC patients remains poor (approximately 25% in 5-years) [1]. Similar to other subtypes of HNSCC, the development of *OSCC* is closely intertwined with behavioral and environmental risk factors, including the consumption of alcohol, tobacco, and betel nut as well as the infection by human papillomavirus [3]–[5]. These risk factors induce proinflammatory cytokine responses, which contribute to the high aggressiveness of malignancy associated with OSCC [6], [7]. A number of cytokines involved in proinflammation are known to be expressed in HNSCCs, including interleukin-1, interleukin-6 (IL-6), interleukin-8 (IL-8), tumor necrosis factor-α, and granulocyte-macrophage colony-stimulating factor [8], [9]. Specifically, the up-regulation of IL-6 and IL-8 has been suggested to play important roles in the development and progression of OSCC and other cancers [10]–[14]. The expression levels of IL-6 and IL-8 have been found to be elevated in the tumor, saliva, and serum of OSCC patients compared to the control groups [15]–[19]. Thus, IL-6 and IL8 has been proposed as potential biomarkers for the diagnosis and follow-up for OSCC patients [10], [12], [14]. However, the cellular regulatory molecules for the modulation of IL-6 and IL-8 responses in OSCC remain to be defined.

G protein-coupled receptors (GPCRs) are critical cell surface proteins involved in modulating inflammatory diseases and cancers [20], [21]. GPCR proteins are a large family of seven-transmembrane domain receptors, which sense external molecules and activate intracellular signal transduction pathways for various physiological responses, including proliferation, differentiation, and chemotaxis. Activation of GPCRs or overexpression of the GPCR signaling molecules are frequently found to play a critical role in promoting tumor growth, metastasis, and angiogenesis [20]–[23]. Most GPCRs transduce signals by activation of heterotrimeric G-proteins that are composed of Gα and Gβ/Gγ subunits. Gα_12_ is defined as one of the four classes of G protein α subunits, functioning in the regulation of cell motility through activating small GTP-binding proteins of Rho family [24]–[26]. Ours and other studies have demonstrated that the activation of Gα_12_ signaling plays a critical role in tumor progression and metastasis of nasopharyngeal carcinoma (NPC) as well as several other human cancers [27]–[29]. However, whether such a relationship also exists in OSCC tumorigenesis has not yet been reported.

Activation of Gα_12_ is known to contribute to inflammatory responses. For instance, Gα_12_ has been shown to mediate the sphingosine 1-phosphate (S1P) induction of cyclooxygenase-2 (COX-2) for the activation of nuclear factor-κB (NF-κB) [30]. Thus, this study was designed to investigate whether Gα_12_ modulates inflammatory responses in promoting tumor invasion in OSCC. Here, we demonstrate that Gα_12_ exerts an impact on proinflammatory cytokine signaling, which in turn contributes to OSCC invasiveness.

## Materials and Methods

### Clinical Samples

All participants provided written informed consent and the study, which was performed in adherence with the Declaration of Helsinki, has been approved by the Institutional Review Board at Chang Gung Memorial Hospital.

### Microarray Analysis

The microarray gene expression data of OSCC tumor samples were retrieved from our previous published transcriptome profiling data of the Affymetrix Exon 1.0 ST array for 57 OSCC tumors and 22 non-cancerous controls [31] (Gene Expression Omnibus database under the accession number GSE25104). For the correlation analysis of gene expression levels and clinical features, we excluded two tumor samples and one control sample because their clinical information was incomplete. The cluster display was generated by Partek software (Partek Inc., Saint Louis, USA) with two-way data clustering. Each row and column represents an individual gene and sample, respectively. Normalized gene expression values were color coded in percentage relative to the mean: blue for values less than the mean and red for values greater than the mean. The association of the expression data with clinicopathological traits, including the presence of ECS (extra-capsular spread), tumor differentiation, pathologic T-status (pathological tumor status), N-status (pathological nodal status), pathological stage, tumor depth and lymphatic invasion, was analyzed by *t-test*. T-status, N-status and pathological stage of the tumors were determined according to the American Joint Committee on Cancer (AJCC). The relative gene expression level was determined using Robust Multiarray Average (RMA), a normalization approach used for normalizing Affymetrix data. The statistical analysis was conducted using R (www.r-project.org) and the SPSS software package 15.0 for Windows (SPSS Inc., Chicago, IL, USA).

To investigate the putative pathways regulated by Gα_12_ in OSCC, Affymetrix Exon 1.0 ST array was used to analyze the transcriptome profile of Gα_12_-depleted OSCC cells. OC-3 and HSC-3 cells transiently transfected with siRNA against Gα_12_ were harvested at 48 h post-transfection. Total RNA was isolated using Qiagen RNeasy Mini Kit (Qiagen, USA) for the array analysis according to the manufacture's standard protocol (Affymetrix, USA). The cluster display and Gene Ontology (GO) enrichment analysis were performed with Partek software (Partek Inc., Saint Louis, USA). The array data have been submitted to Gene Expression Omnibus database and are publically available under the accession number GSE44111.

### Cell Culture

Four human OSCC cell lines, HSC-3, SCC25, OC-3 and CGHNC9 were used in this study. HSC-3 (JCRB0623) cells were originally obtained from the JCRB cell bank (Osaka, Japan). SCC25 (ATCC® CRL-1628™) cell line was purchased from Food Industry Research and Development Institute in Taiwan. OC-3 and CGHNC9 cells were originally established in Taiwan and authenticated using the experiments described before [32], [33].

HSC-3 was cultured in MEM (Invitrogen, USA), SCC25 in 1∶1 DMEM/F12 (Invitrogen, USA), OC-3 in 1∶1 DMEM/KSFM (keratinocyte serum-free medium) (Invitrogen, USA) and CGHNC9 in DMEM (Invitrogen, USA). All culture media were supplemented with 10% fetal bovine serum (Invitrogen, USA) and 50 units/ml penicillin and streptomycin (Invitrogen, USA). Cells were maintained in a 37°C incubator with 5% CO_2_.

### Cell Transfection

To knock down Gα_12_, OSCC cells were seeded at a density of 5×10^4^ per well in a 24-well culture plate 24 h before transfection with Gα_12_-siRNA (siGα_12_) or a non-targeting control siRNA (siCtrl) at a concentration of 50 nmol/L using DharmaFECT transfection reagents (Dharmacon, USA). To overexpress Gα_12_, OSCC cells were transiently transfected with Gα_12_ expression plasmids (pcDNA3-Gα_12_) or a mock control plasmid (pcDNA3) using Lipofectamin 2000 transfection reagents (Invitrogen, USA). The pcDNA3-Gα_12_ plasmid was obtained from the Missouri S&T cDNA Resource Center as previously described [27]. For functional assays, cells were collected at 1–3 days post-transfection.

### Quantitative real-time Reverse Transcription-PCR (qPCR)

For reverse transcription of cellular mRNA to cDNA, an input of 2 µg of total RNA was used for the High-capacity cDNA Reverse transcription Kits (ABI Applied Biosystems, USA) according to the manufacturer's instructions. qPCR was performed on a Step One Real-time PCR system (ABI Applied Biosystems, USA) by using the KAPA SYBR FAST qPCR Kits (KAPA Biosystems, USA). The primer sequences of Gα_12_ used for qPCR were: forward-GTTTGTCGTCGTTGAGC, reverse-AGTAGTTTCACTCGCCC; for IL-8: forward-GGAGTGCTAAAGAACTTAGATG, reverse-TGGGGTCCAGACAGAG; for IL-6: forward-CAAAGATGTAGCCGCCC, reverse-GTTCAGGTTGTTTTCTGCC; for GAPDH: forward-CCTGCCAAATATGATGACATCAAG, reverse-ACCCTGTTGCTGTAGCCAAA.

Total RNA was purified using the Illustra RNAspin Mini RNA Isolation Kits (GE healthcare, USA) according to the manufacturer's instructions. The amount of target transcript was estimated by the respective standard curves and normalized to the amount of GAPDH transcript. The results were expressed as a relative fold change to the control. For semi-quantitative RT-PCR, the number of cycles for IL-6 and IL-8 was individually optimized. PCR products were analyzed on a 2% agarose gel.

### Immunohistochemistry

To determine the protein expression level of Gα_12_, immunohistochemical staining of OSCC tissue sections was conducted as described previously [27] using anti-Gα_12_ primary antibody (1∶75; sc-409; Santa Cruz Biotechnology). The images of Gα_12_ staining were captured by a Leica DM2500 Upright Fluorescence Microscope at 20× objective.

### Western Blotting

The harvested cells were washed twice with cold PBS buffer and lysed as described previously [27]. The total protein concentration was measured using the Bio-Rad Protein Assay (Bio-Rad, USA). Equal amounts of cell lysates were separated by SDS-PAGE and transferred to polyvinylidene fluoride membranes. The membrane was blocked and blotted with indicated primary antibodies: anti-Gα_12_ (Santa Cruz Biotechnology, USA), anti-GAPDH (glyceraldehyde-3-phosphate dehydrogenase) (Lab Frontier, USA). After washing, the membrane was incubated with HRP–conjugated secondary antibody (Jackson ImmunoResearch Laboratories, USA) and developed with enhanced chemiluminescence detection reagents (PerkinElmer, USA).

### Migration and Matrigel Invasion Assay

OSCC cells transfected with indicated expression plasmids or siRNAs were harvested at 48 h post-transfection. Invasion capacity was analyzed in a Boyden chamber consisting of cell culture inserts with an 8 µm pore-sized PET membrane coated with matrigel (BD Biosciences, USA) according to the manufacturer's instructions. For the cell migration assay, the chamber without matrigel coating was used. After seeding cells into the chamber, cells were incubated for 16 h or 24 h at 37°C for migration or invasion, respectively. The recombinant human IL-6 was obtained from PeproTech (Rocky Hill, NJ). The neutralizing IL-6 and IL-8 antibodies and recombinant human IL-8 were obtained from R&D Systems (Minneapolis, MN). Migrated or invaded cells were stained with 0.1% crystal violet in 1% formaldehyde and 20% ethanol. The number of invaded cells was counted at least in five distinct fields for each duplicate chamber. The results were expressed as a relative percentage to control cells.

### IL-6 and IL-8 ELISA

OSCC cells were seeded into 12-well plates (1×10^5^ cells/well) and cultured overnight prior to the transfection of siRNA or plasmid DNA. At 24 h post-transfection, cells were transferred to serum-free medium for 24 h before being assayed for IL-6 and IL-8 protein levels in medium by ELISA. For the LPA treatment, cells at 24 h post-transfection were incubated in 10 µM LPA-containing (Sigma-Aldrich, USA) serum-depleted medium for 12 h. The conditioned media were centrifuged to remove cell debris for ELISA assays using DuoSet ELISA Development kit (R&D Systems, USA) according to the manufacturer's protocols. The quantification data were expressed as a relative fold change to control group.

## Results

### Gα_12_ is Significantly Up-regulated in OSCC Patients and Correlates with Extra-capsular Spread

**Figure 1:**
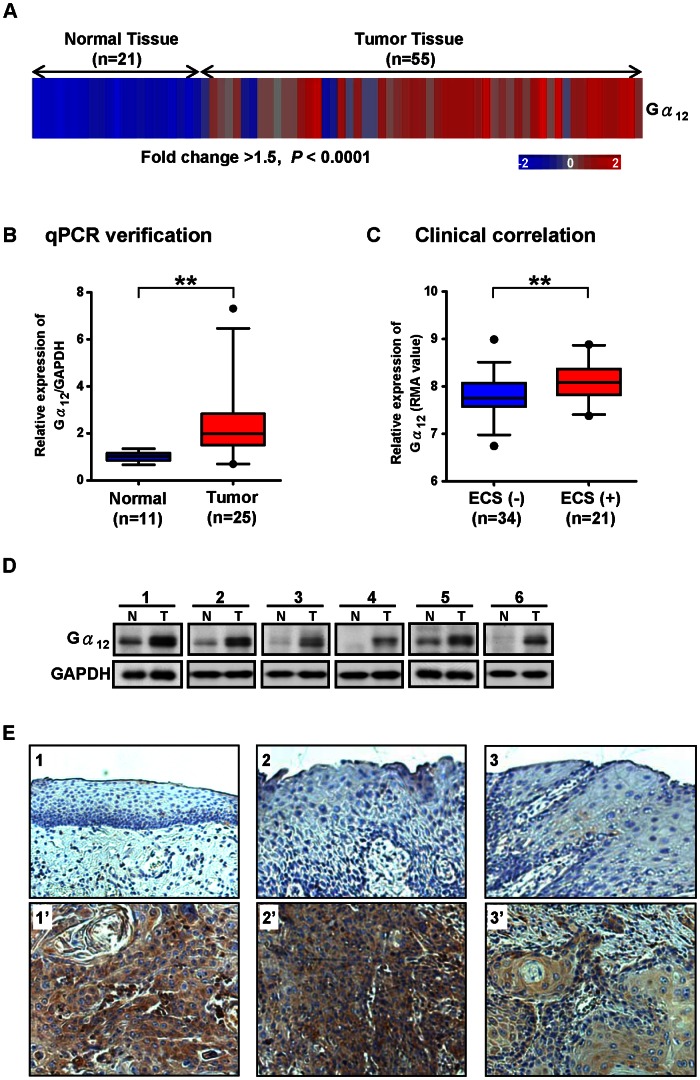
The up-regulation of Gα_12_ in OSCC patients correlates with Extra-capsular spread. (A) The Gα_12_ expression is significantly up-regulated in 55 OSCC tumors compared to 21 normal control tissues (fold change >1.5, *P*<10^−10^). The microarray data was analyzed by two-way clustering. Each column represents an individual clinical sample. Normalized gene expression values were color coded in percentage relative to the mean: blue for values less than the mean and red for values greater than the mean. (B) Quantitative RT-PCR (qPCR) analysis of Gα_12_ in 25 OSCC tumors compared to 11 normal mucosa tissues. The results were normalized to GAPDH expression levels and then analyzed by *t-test*, **P<0.01. Box plots display the median, 25th and 75th percentiles. Whiskers represent 5–95 percentiles and dots the outliers. (C) The box plot shows the relative gene expression values (RMA, log2) of Gα_12_ for extra-capsular spread (ECS) positive (+) and negative (−) patients. Statistical results were analyzed by *t-test*, **P<0.01. (D) Western blot analysis of Gα_12_ levels in 6 paired samples of OSCC and adjacent normal/pre-cancerous tissues. The Gα_12_ protein levels were found to be markedly up-regulated in OSCC tumor tissues compared to the GAPDH loading control. (E) Representative immunohistochemical images for Gα_12_ staining patterns in the paraffin-embedded section of OSCC biopsies. Gα_12_ immunoreactivity was detected primarily in the membrane and cytoplasm of OSCC (lower panel). In contrast, the adjacent normal and pre-cancerous oral tissues of individual patients showed very low immunoreactivity (upper panel). Original magnification, ×200.

Our previous study of NPC has shown that Gα_12_ gene expression is up-regulated in tumor cells, and is also important in facilitating tumor invasiveness [27]. Thus, it was of interest to investigate whether Gα_12_ also played a role in the tumorigenesis of OSCC, a specific subtype of HNSCC. By analyzing our previous transcriptome profiling data for OSCC [31] (Gene Expression Omnibus database under the accession number GSE25104), we characterized the gene expression pattern of Gα_12_ in OSCC and normal mucosa tissues. The array results showed that Gα_12_ was significantly up-regulated in 55 OSCC tissues compared to 21 normal controls (*P*<10^−10^ with fold change cutoff of >1.5) (Figure 1A). To validate the array results, we performed quantitative RT-PCR (qPCR) analysis on 25 OSCC and 11 normal mucosa tissues. As suspected, Gα_12_ was significantly up-regulated in OSCC tissues compared to controls (*P* = 0.0036) (Figure 1B). Because the elevation of Gα_12_ expression is highly associated with tumor invasiveness in several human cancers [27]–[29], we examined the correlation between the Gα_12_ transcription level and clinicopathological characteristics, including tumor differentiation, pathologic T-status, N-status, pathological stage, tumor depth, ECS, and lymphatic invasion in 55 OSCC patients. We found evidence suggesting a significant correlation between higher Gα_12_ levels and ECS (*P* = 0.009) (Figure 1C). There appeared to be no discernible correlation between Gα_12_ and the other clinicopathological characteristics (data not shown). Since ECS is a known prognostic factor of tumor aggressiveness in HNSCC, including high loco-regional recurrence rates, distant metastasis, and poor prognosis [34]–[37], the correlation of Gα_12_ levels with ECS may suggest a role of Gα_12_ in OSCC invasiveness. To determine whether the Gα_12_ protein levels were also elevated in OSCC tumors as shown in its gene expression, we measured the Gα_12_ protein level in OSCC tumors by Western blotting and immunohistochemistry. Western blot analysis of OSCC tumor tissues revealed that Gα_12_ was markedly up-regulated compared to controls (Figure 1D). The results of immunohistochemical analysis also confirmed that of Western blot analysis. The representative micrographs of Gα_12_ staining showed clear immunoreactivity of Gα_12_ in most OSCC tumors (18 of the 20 cases, 90%) but the signal was either absent or weak in adjacent normal or precancerous tissues (Figure 1E). These results suggest that Gα_12_ expressions are up-regulated in OSCC tumors, and may be associated with tumor invasiveness.

### Gα_12_ Modulates Cell Migration and Invasion Abilities of OSCC Cells

**Figure 2:**
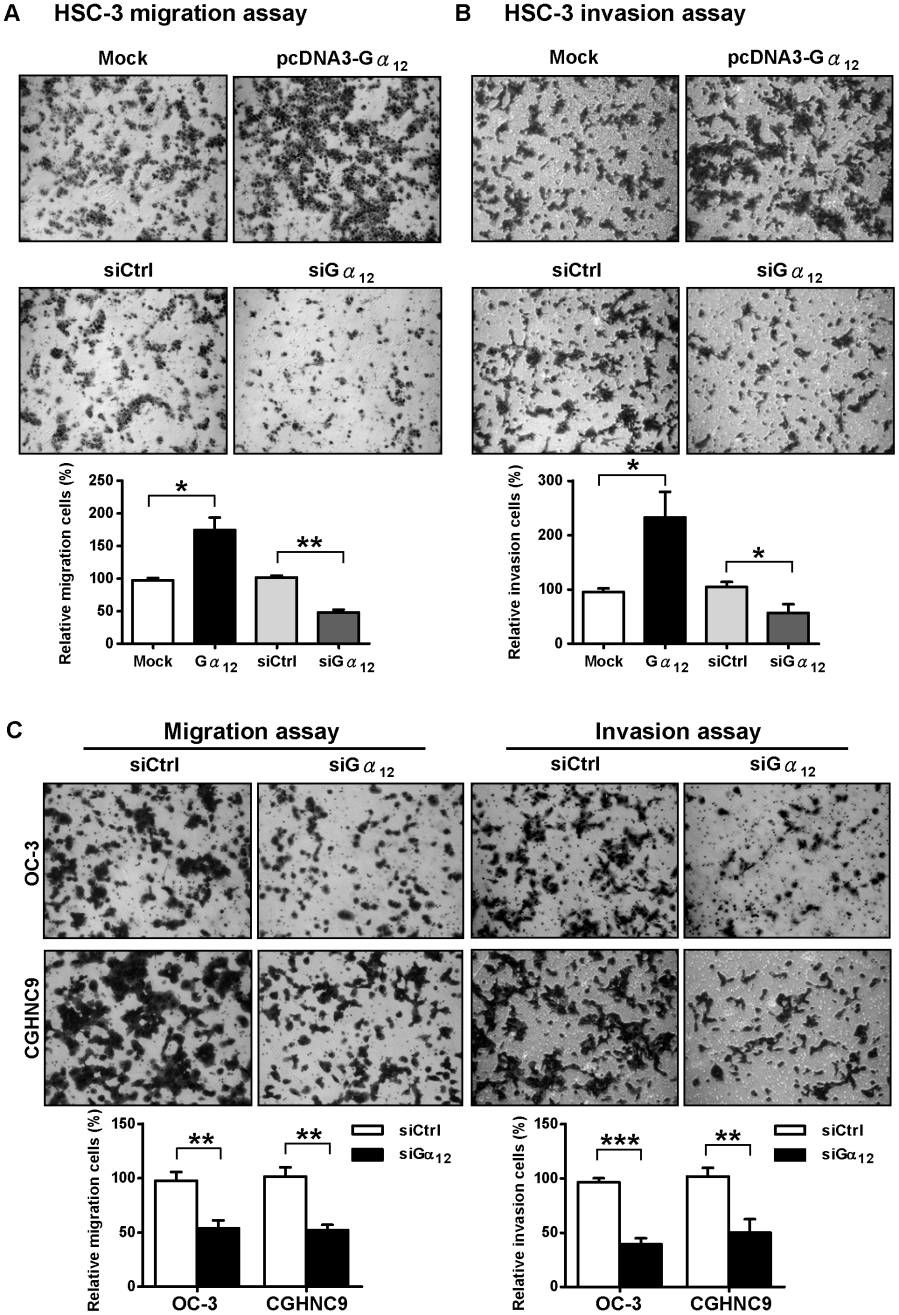
Gα_12_ promotes OSCC cell migration and invasion. (A) The transwell migration assay of Gα_12_ overexpressed (Gα_12_), or Gα_12_ depleted (siGα_12_) HSC-3 cells stained with crystal violet. The lower panel shows the quantitative results by three independent experiments. (B) The transwell invasion assay of Gα_12_ overexpressed (Gα_12_), or Gα_12_ depleted (siGα_12_) HSC-3 cells. The lower panel shows the quantitative results by three independent experiments. (C) Depletion of Gα_12_ in two other OSCC cell lines (OC-3 and CGHNC9) also decreased cell migration and invasion. The knockdown efficiency and overexpression level of Gα_12_ in four different OSCC cell lines used in this study are demonstrated in Figure S1. The bottom panel shows the quantitative results. All the quantitative values were calculated at least in five distinct fields of each chamber. The data are expressed as a relative percentage to the controls. The statistic results were analyzed by *t-test*, *P<0.05, **P<0.01, ***P<0.001. Error bars represent the standard deviation (SD) of the mean from three independent experiments.

To validate the role of Gα_12_ in facilitating the invasive behavior of OSCC cells, we conducted transwell migration and invasion assays in cells overexpressing or depleted Gα_12_. The knockdown efficiency and overexpression level of Gα_12_ in these cell lines were demonstrated in Figure S1. As expected, the cell migration and invasion ability of OSCC tumor cells (HSC-3) was significantly increased by Gα_12_ overexpression and decreased by RNAi knockdown (Figure 2A and B). To further validate these results, we also depleted Gα_12_ by siRNA in two other OSCC cell lines (OC-3, and CGHNC9) and determined the effect on cell invasiveness. Results showed that the depletion of Gα_12_ also suppressed tumor cell migration and invasion ability in these two other OSCC cell lines (Figure 2C). Taken together, these data support the idea that Gα_12_ promotes the invasive behavior of OSCC cells.

### Comparative Transcriptome Analysis Reveals the Enrichment of Immune-related Pathways in the Gα_12_-depleted OSCC Cells

**Figure 3:**
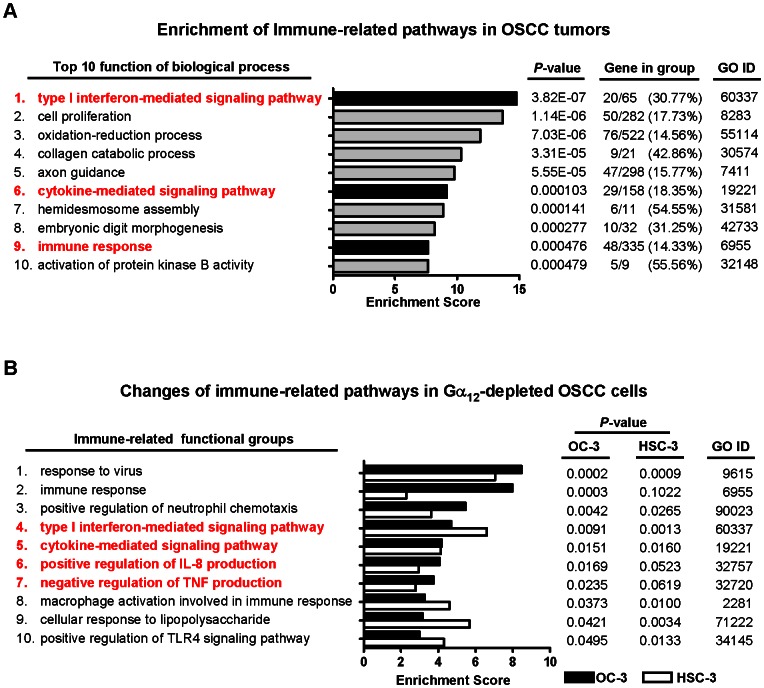
Transcriptome analysis reveals changes of immune-related pathways in OSCC and in Gα_12_-depleted OSCC cell lines. (A) Comparative transcriptome analysis of OSCC tumors reveals that cytokine and other immune-related functional groups are listed in the top ten GO terms. A total of 1,616 differently expressed genes selected by 1.5 fold change cut-off (positive false discovery rate *q*<10^−8^) in 55 OSCC tumors compared to 21 normal control tissues were analyzed by GO and pathway analysis tools. Functional groups of the inflammation-related pathways are highlight in red. (B) The immune-related signaling pathways are significantly impaired in the Gα_12_-depleted OC-3 and HSC-3 cell lines. An arbitrary 2.0 fold-change cut-off is used to filter the differentially expressed genes compared between Gα_12_-depleted and non-targeted siRNA control cells for the GO enrichment analysis. A total of 58 genes for HSC-3 cells and 218 genes for OC-3 cells were subjected to the analysis. The cytokine and interferon-mediated pathways (highlighted in red) were found in the GO terms for both cell lines. Detailed information of the GO terms is shown in Table S1.

Several reports have suggested that the immune-related genes may serve as potential biomarkers or therapeutic targets for OSCC [6], [12], [19], but the underlying mechanisms still remained to be further investigated. We conducted gene ontology (GO) analysis for our previous transcriptome data of OSCC tumors to better understand the biological characteristics of the differentially expressed genes in OSCC. As noted by previous studies [12], [19], [38], cytokine and other immune-related signaling pathways were found to be highly enriched in the top ten GO terms (Figure 3A). Although GPCR signaling is important for the inflammatory response and invasiveness of cancers, it remains to be defined whether Gα_12_ is required for the inflammation-associated tumorigenesis in OSCC. To test this hypothesis, we performed microarray analysis for the Gα_12_-depleted HSC-3 and OC-3 OSCC cells. The differentially expressed genes were selected by an arbitrary fold-change cut-off of 2.0 for GO enrichment analysis. Similar to that found in OSCC clinical samples, comparative transcriptome analysis revealed that many immune-related functional groups, including cytokine and interferon-mediated pathways, were significantly changed in both cell lines (Figure 3B; for detail GO terms see Table S1), suggesting that Gα_12_ may play a critical role in modulating inflammatory cytokine responses during OSCC tumorigenesis.

### Gα_12_ Correlates with IL-6 and IL-8 Expression Levels in OSCC Patients and Stimulates IL-6 and IL-8 Expressions in OSCC cell Lines

**Figure 4:**
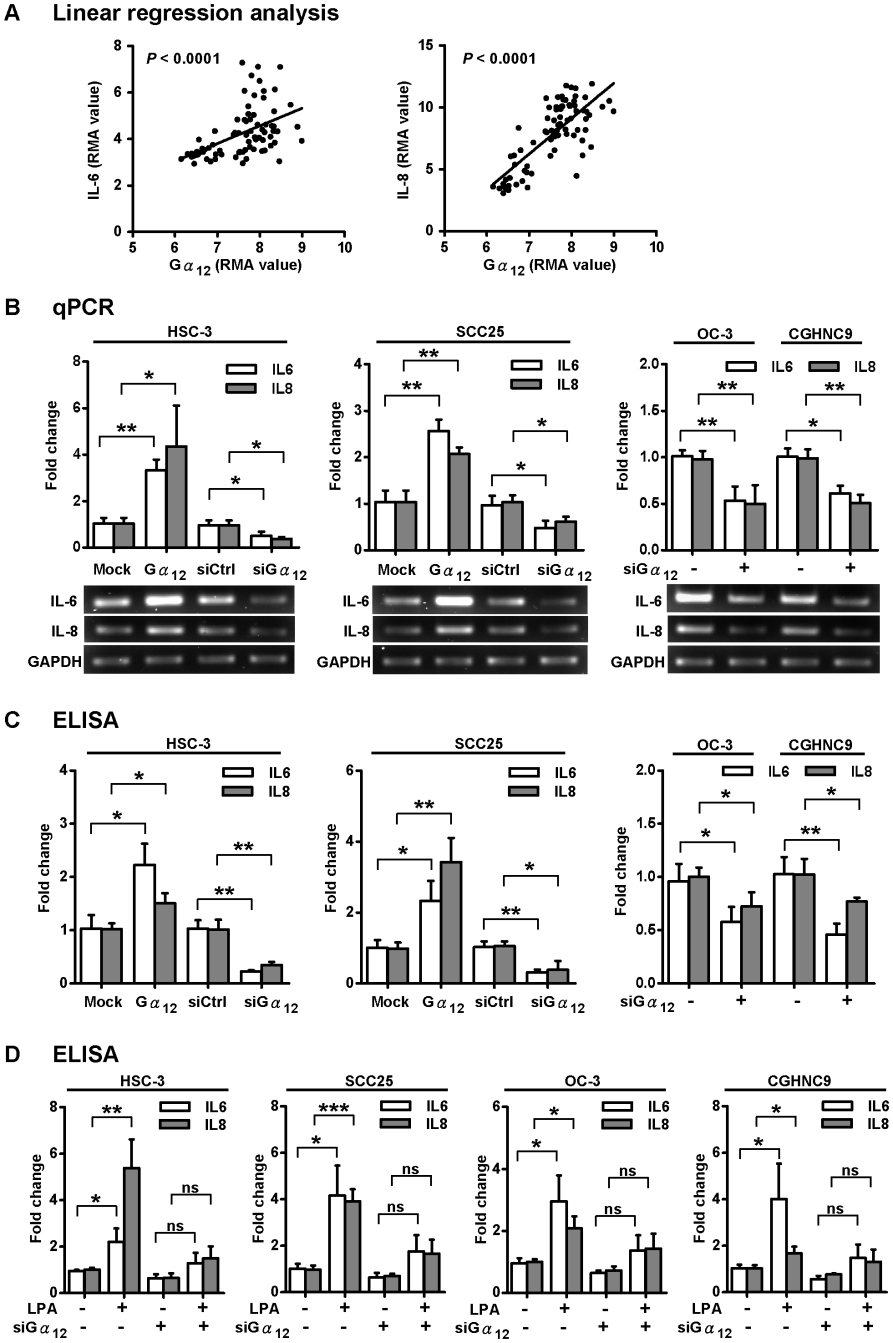
Gα_12_-dependent regulation of IL-6 and IL-8 in OSCC. (A) Dot plots of the linear regression analysis showing a positive correlation of gene expressions between Gα_12_ and IL-6/IL-8 in OSCC tumors and normal mucosa tissues. The relative expression scales are shown by RMA value in the microarray data. (B) IL-6 and IL-8 mRNA levels are positively regulated by Gα_12_ in OSCC cells. Gα_12_ levels in four different OSCC cell lines (HSC-3, SCC25, OC3, and CGHNC9) were altered by the transient overexpression or RNAi knockdown of Gα_12_. The qPCR results were normalized against GAPDH. The lower panel shows the electrophoresis image of the RT-PCR products. (C) The secreted proteins of IL-6 and IL-8 are up-regulated by Gα_12_ in OSCC cells. ELISA assay was used to measure IL-6 and IL-8 in the conditioned media of the Gα_12_-overexpressing or -depleted HSC-3, SCC25, OC-3, and CGHNC9 cells. (D) The LPA-induced IL-6 and IL-8 production is regulated by Gα_12_. IL-6 and IL-8 protein levels in conditioned media of OSCC cells were measured by ELISA assay. The baseline IL-6 and IL-8 levels in conditioned media from four different OSCC cell lines are shown in Figure S2. All the quantitative results are expressed as a fold change relative to the controls. The statistical results were analyzed by *t-test*, *P<0.05, **P<0.01, ***P<0.001. “ns” means no significance. Error bars represent SD of the mean from three independent experiments.

Since our transcriptome analysis of OSCC suggested that IL-6 and IL-8 cytokines were significantly up-regulated in OSCC patients (*P*<0.0001, with fold change 2.5 and 16.1 respectively), we hypothesized that these cytokine responses were associated with the up-regulation of Gα_12_. To test this idea, we analyzed the microarray data for the differential expression of genes that correlated with increased Gα_12_ levels in OSCC patients. Indeed, the expression levels of IL-6 and IL-8 were significantly correlated with the levels of Gα_12_ in a total of 76 OSCC and normal tissues (*P*<0.0001) (Figure 4A), suggesting that Gα_12_ might associate with the production of IL-6 and IL-8 in OSCC. To test this hypothesis, we investigated whether altering the Gα_12_ levels by gene silencing or transient overexpression would affect the IL-6 and IL-8 expression levels in OSCC cells. Results from studies in HSC3 and SCC25 cell lines demonstrated that the IL-6 and IL-8 levels were increased and decreased by the overexpression and depletion of Gα_12_, respectively (Figure 4B, left and middle panels). Similarly, we depleted the Gα_12_ expressions in OC3 and CGHNC9 cell lines and found that both the IL-6 and IL-8 gene expression levels were significantly decreased (Figure 4B, right panel). Additionally, we examined whether secreted cytokines in the culture supernatants were also affected in accordance with their mRNA expressions by the change of Gα_12_ levels. The ELISA assays of secreted cytokines from HSC-3 and SCC25 cells showed that the IL-6 and IL-8 protein levels were also increased and decreased by the overexpression and RNAi knockdown of Gα_12_, respectively (Figure 4C, left and middle panels). Depletion of Gα_12_ in OC3 and CGHNC9 cell lines (with high baseline levels of IL-6 and IL-8; see Figure S2) resulted in the decrease of IL-6 and IL-8 protein levels (Figure 4C, right panel). These results suggest that Gα_12_ is a critical modulator in stimulating the production of IL-6 and IL-8 in OSCC cells. To further substantiate this view, we treated the OSCC cells with lysophosphatidic acid (LPA) to investigate the role of Gα_12_ in modulation of IL-6 and IL-8 responses. LPA has been previously shown to promote cell proliferation and migration via stimulation of Gα_12_ signaling [39], [40] and can also elicit IL-6 and IL-8 production in various human cell types [41]–[43]. Indeed, our results showed that LPA stimulated IL-6 and IL-8 secretion into culture media from four different OSCC cell lines (HSC3, SCC25, OC3, and CGHNC9) but RNAi depletion of Gα_12_ suppressed the LPA-induced cytokine expressions (Figure 4D). Together, these results strongly suggest that the up-regulated Gα_12_ is a critical stimulus for the pro-inflammatory cytokine responses in OSCC.

### The role of Gα_12_ in the Stimulation of Cell Invasiveness Requires IL-6 and IL-8 in OSCC Cells

**Figure 5:**
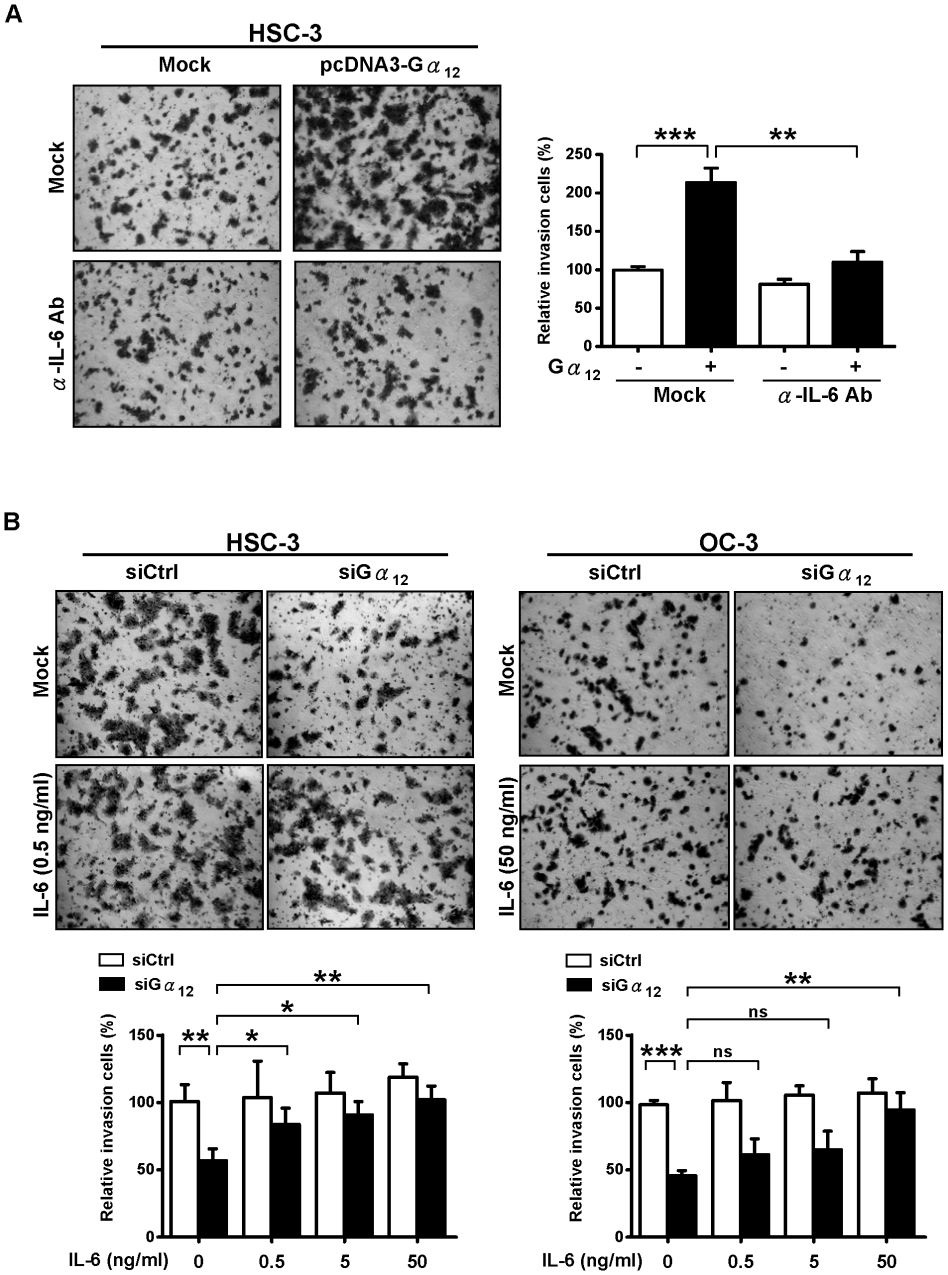
Gα_12_ promotes OSCC cell invasive behavior via the regulation of IL-6. **(A)** The Gα_12_-induced cell invasion of HSC-3 is suppressed by the treatment of cells with anti-IL-6 antibody. Representative images of the transwell invasion assays of the Gα_12_-overexpressing HSC-3 cells treated with or without anti-IL-6 antibody for 24 h (left panel). Quantification of invasion is shown in the right panel. (B) Supplementation of culture media with IL-6 restores cell invasion in Gα_12_-depleted cells. The Gα_12_-depleted HSC-3 and OC-3 cell lines were treated with or without three different concentrations of recombinant human IL-6 (0.5 ng/ml, 5 ng/ml, and 50 ng/ml) for 24 h prior to transwell invasion assays. The quantitative results of three independent experiments were analyzed by *t-test*. *P<0.05, **P<0.01, ***P<0.001. “ns” means no significance. Error bars represent SD of the mean from three independent experiments.

Although we have demonstrated the role of Gα_12_ in promotion of OSCC cell invasive behavior and pro-inflammatory cytokines expressions, we have not yet established the functional link between the Gα_12_-dependent cytokine response and cell invasiveness. To test whether the induction of proinflammatory cytokines was required for the promotion of tumor cell invasion, we neutralized the endogenous IL-6 in the Gα_12_-overexpressed HSC-3 cells and examined the tumor cell invasiveness via transwell invasion assays. Results showed that the anti-human IL-6 antibody, as compared to IgG control, significantly suppressed the cell invasion (about 80%) in cells transiently overexpressed with Gα_12_ (Figure 5A). To further test this idea, we first decreased the tumor cell invasiveness by depleting the endogenous Gα_12_, and then introduced recombinant IL-6 into the culture medium to determine if the cell invasiveness could be resumed by IL-6. The transwell invasion assays in HSC-3 and OC-3 cells suggested that the tumor cell invasiveness was restored by IL-6 (Figure 5B).

Moreover, we also tested whether antibody neutralization of IL-8 suppresses the cell invasiveness enhanced by overexpression of Gα_12_. By using the anti-IL-8 antibody to neutralize IL-8 in HSC-3 and SCC25 cells overexpressing Gα_12_, we examined if the migration ability of the tumor cells was decreased. The results showed that the cell migration ability was significantly diminished by neutralizing the endogenous IL-8 in both HSC-3 and SCC25 cells (Figure S3), suggesting that IL-8 functions, similar to IL-6, as an important downstream effector of Gα_12_ signaling in regulating OSCC cell invasive behavior. To further substantiate this idea, we tested whether supplementation of recombinant IL-8 restores the migration ability decreased by the depletion of Gα_12_ in OSCC cells. As expected, the decreased migration ability of HSC-3 cells was restored by the recombinant IL-8 (Figure S4). Together, these results indicate that the Gα_12_ induction of cell invasiveness is regulated by the proinflammatory cytokine**s** IL-6 and IL-8 in OSCC cells.

## Discussion

Dysregulation of inflammatory pathways during tumorigenesis contributes to the invasive characteristics of cancer; therefore the associated mediators of these pathways become favorable targets in the search for new pharmacological medications. As in many other cancers, the development and invasiveness of OSCC is intimately related to chronic inflammation [6]–[9]. However, the signaling cascade that leads to the proinflammatory response has been poorly understood. In this study, we found evidence to support that the increase of Gα_12_ is an important stimulator for the production of proinflammatory cytokines IL-6 and IL-8 and tumor invasiveness in OSCC. We further showed that the Gα_12_-induced cell invasiveness was mediated through the regulation of IL-6 and IL-8 in OSCC.

Gα_12_ is known to regulate several cellular processes through the modulation of transcription factors, including NF-κB and AP-1 [23], [44]. For instance, it has been demonstrated that the coupling of Gα_12_ to S1P receptors induces COX-2 expression via NF-κB [30]. Since the S1P producing enzyme-sphingosine kinase 1 (SPHK1) has been reported to be up-regulated in HNSCC [45], it is possible that the S1P-NF-κB signaling might modulate the Gα_12_ induction of cytokine responses. Supporting this idea, our preliminary data showed that the S1P treatment of OSCC cells induced IL-6 and IL-8 expressions, but the RNAi-mediated depletion of Gα_12_ abolished the S1P induction of IL-6 and IL-8 (unpublished data). Future work will aim to test whether disruption of NF-κB diminishes the Gα_12_-induced cytokine expressions. The possible GPCRs that couple Gα_12_ in stimulating IL-6/IL-8 production in OSCC tumors also remain to be identified in the future.

In addition to the role in promoting tumor cell invasiveness, we speculate that the Gα_12_ induction of IL-6 and IL-8 production might also be involved in the drug resistance of OSCC, because several cytokines, including IL-6 and IL-8, are known to contribute to drug resistance in several different cancer types [46]–[48]. Drug resistance remains a critical hurdle for the success of cancer chemotherapy. Although chemotherapy is currently the primary treatment for both resectable and advanced OSCC patients, it has been reported to only marginally increase the survival rate for those patients [2], [3], [49]. Thus, it is important to investigate whether Gα_12_ signaling is linked to the drug resistance of OSCC. We are currently assessing if Gα_12_ could be a potential biomarker for the prediction of anti-cancer drug resistance by analyzing the correlation of Gα_12_ to the patients' responses to chemotherapy.

Another issue concerns the complex interplay between Gα_12_ and inflammatory cytokines. Although our data strongly suggests that Gα_12_ promotes inflammatory cytokines production in OSCC, we cannot exclude the possibility that the interplay is bidirectional or reciprocal because proinflammatory cytokines have been shown to transactivate S1P receptors via stimulation of S1P production, leading to the activation of G protein signaling [50]. Thus, it will be of interest in the future to investigate whether the increased cytokines inversely modulate the Gα_12_ signaling in OSCC.

Currently, due to the high invasiveness of OSCC, very few promising pharmacologic therapeutics have been developed and backed by clinical trials. Because Gα_12_ is involved in the inflammatory invasion of OSCC, we believe it can potentially become a useful therapeutic target or biomarker for the invasive OSCC, though further clinicopathological delineation of the association of Gα_12_ and IL-6/IL-8 functions are needed before Gα_12_ becomes an eligible prognostic marker for this disease.

## Supporting Information

Table S1 List of immune-related functional groups impaired in the Gα_12_-depleted OSCC cells.
(TIF)

Figure S1 **Western blot analysis of siRNA knockdown efficiencies and overexpression levels of Gα_12_ in four different OSCC cell lines (HSC-3, SCC25, OC-3, and CGHNC9).**
(TIF)

Figure S2 **Baseline levels of IL-6 and IL-8 secreted in conditioned media from four different OSCC cell lines (HSC-3, SCC25, OC-3, and CGHNC9).** Quantitative measurements of IL-6 and IL-8 were determined by ELISA assays.
(TIF)

Figure S3 **IL-8 neutralizing antibody abolishes the Gα_12_-induced OSCC cell migration.** (A), (B) Transwell migration assays of the Gα_12_-overexpressing HSC-3 and SCC25 cells treated with neutralizing antibody (10 µg/ml) against IL-8 for 16 h. Quantification of migration is shown in the right panel. Error bars represent SD of the mean from three independent experiments and analyzed by *t-test*. *P<0.05, **P<0.01.
(TIF)

Figure S4 **Recombinant IL-8 restores the migration ability reduced by transiently depleted Gα_12_ in HSC-3 cells.** Representative images show the migration of the Gα_12_-depleted HSC-3 cells through transwells. Cells treated with or without recombinant human IL-8 (1 ng/ml) for 16 h. Quantification of migration is shown in the right panel. Error bars represent SD of the mean from three independent experiments and analyzed by *t-test*. *P<0.05.
(TIF)
