# Supplementary figures and images for: Gα12 Drives Invasion of Oral Squamous Cell Carcinoma through Up-Regulation of Proinflammatory Cytokines
Source: PLoS One. 2013 Jun 7;8(6):e66133. doi: 10.1371/journal.pone.0066133 (PMC3676329; doi:10.1371/journal.pone.0066133)

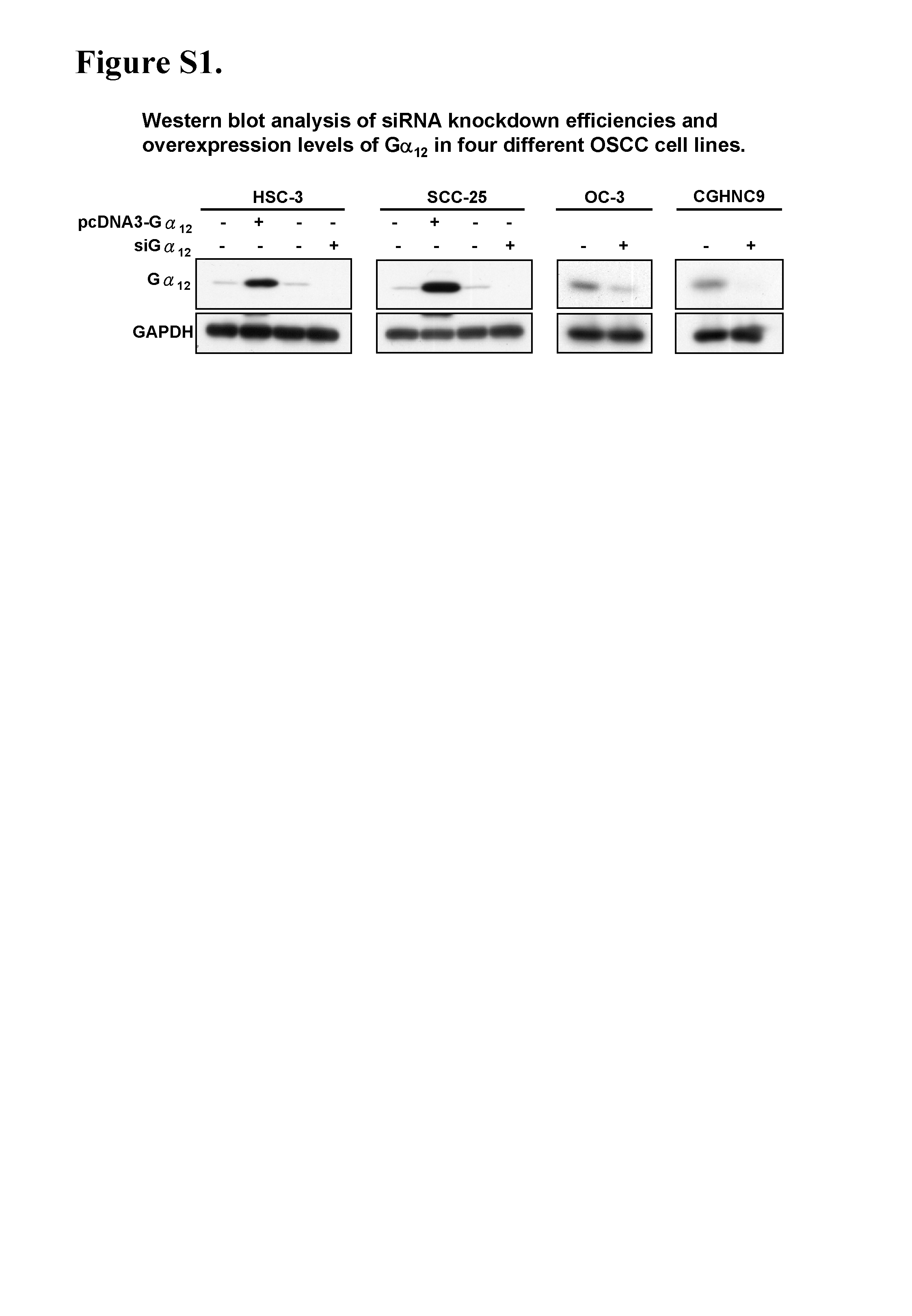

Supplement: Figure S1 — Western blot analysis of siRNA knockdown efficiencies and overexpression levels of Gα12 in four different OSCC cell lines (HSC-3, SCC25, OC-3, and CGHNC9). (TIF) [file pone.0066133.s002.tif]

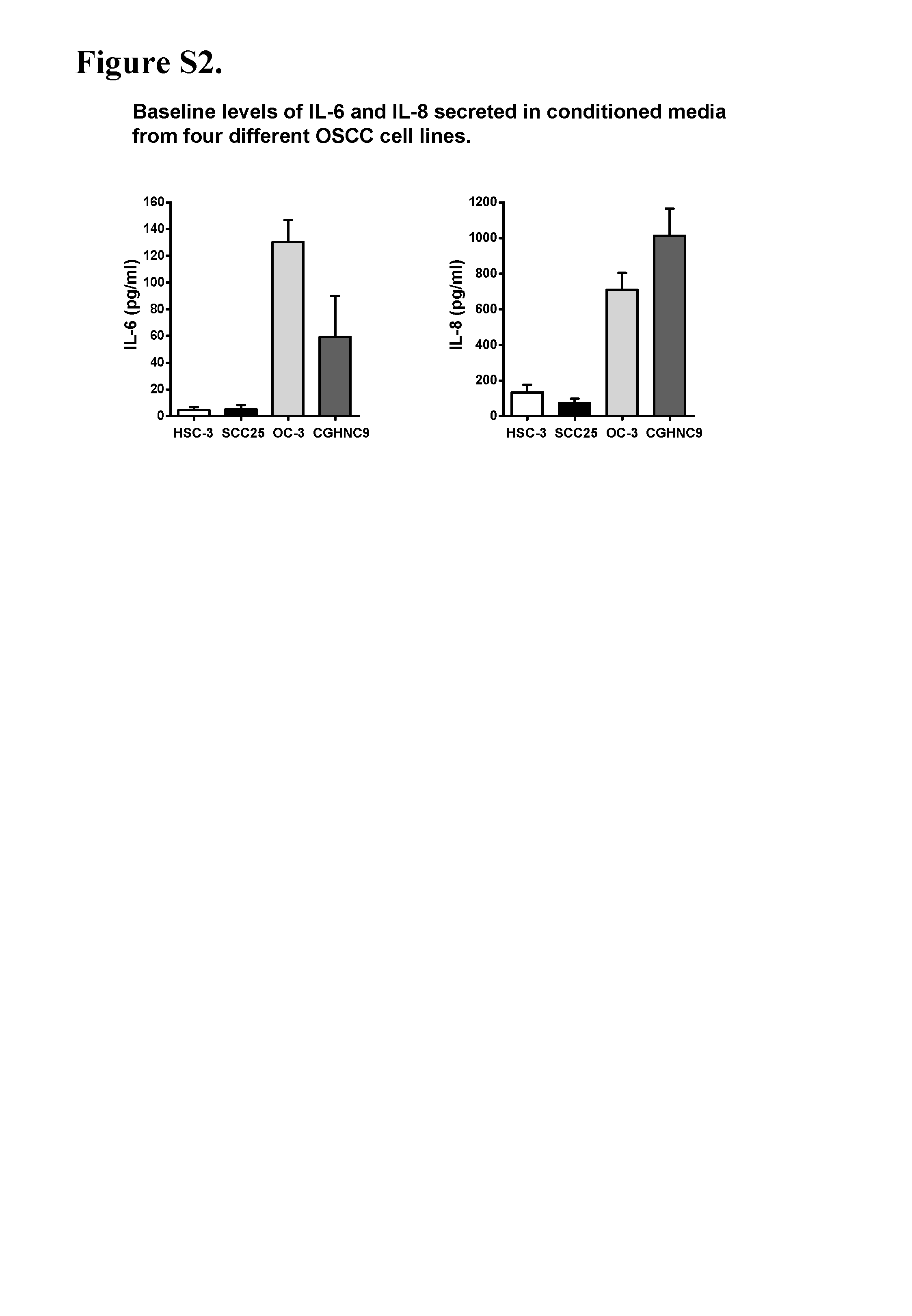

Supplement: Figure S2 — Baseline levels of IL-6 and IL-8 secreted in conditioned media from four different OSCC cell lines (HSC-3, SCC25, OC-3, and CGHNC9). Quantitative measurements of IL-6 and IL-8 were determined by ELISA assays. (TIF) [file pone.0066133.s003.tif]

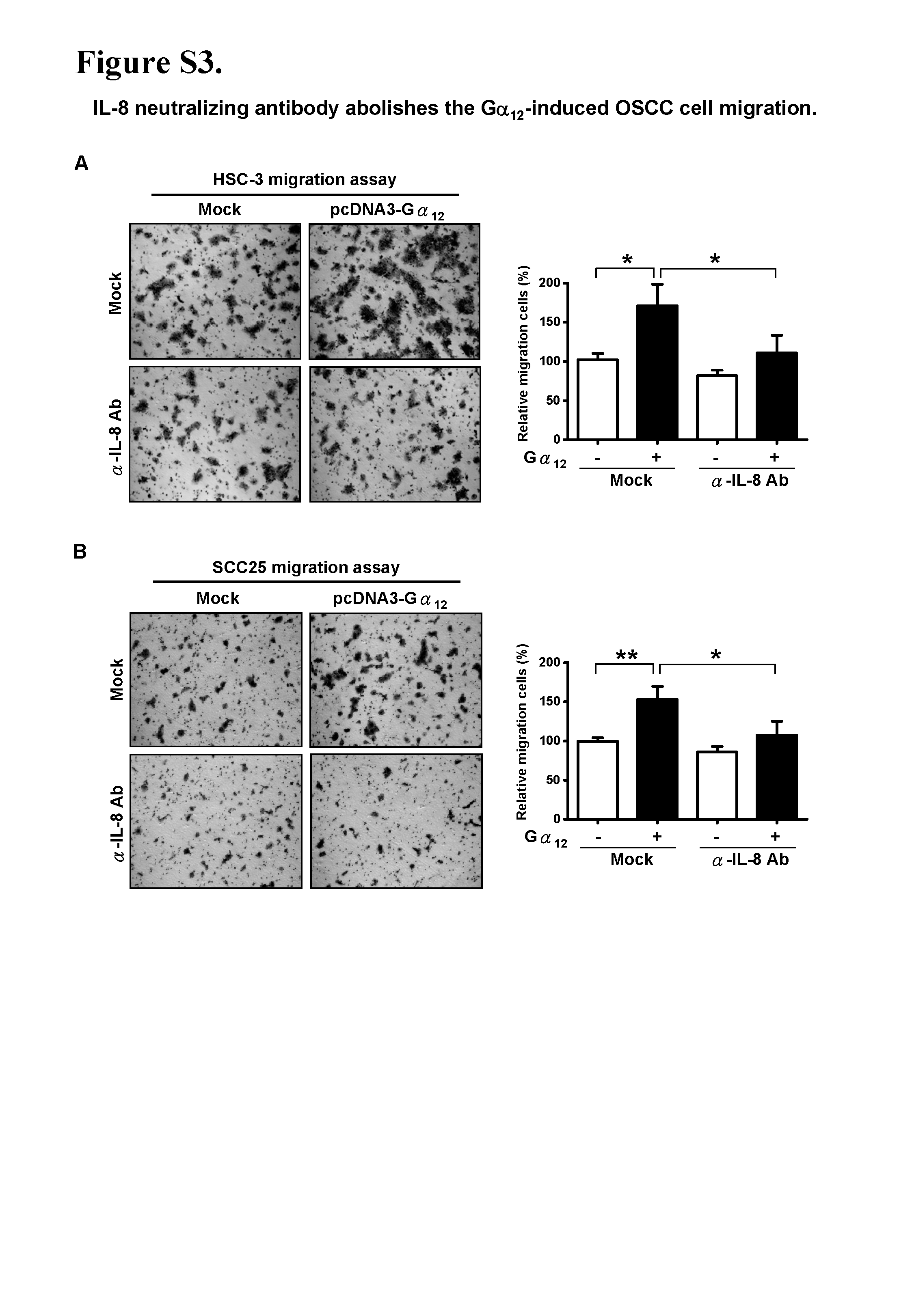

Supplement: Figure S3 — IL-8 neutralizing antibody abolishes the Gα12-induced OSCC cell migration. (A), (B) Transwell migration assays of the Gα12-overexpressing HSC-3 and SCC25 cells treated with neutralizing antibody (10 µg/ml) against IL-8 for 16 h. Quantification of migration is shown in the right panel. Error bars represent SD of the mean from three independent experiments and analyzed by t-test. *P<0.05, **P<0.01. (TIF) [file pone.0066133.s004.tif]

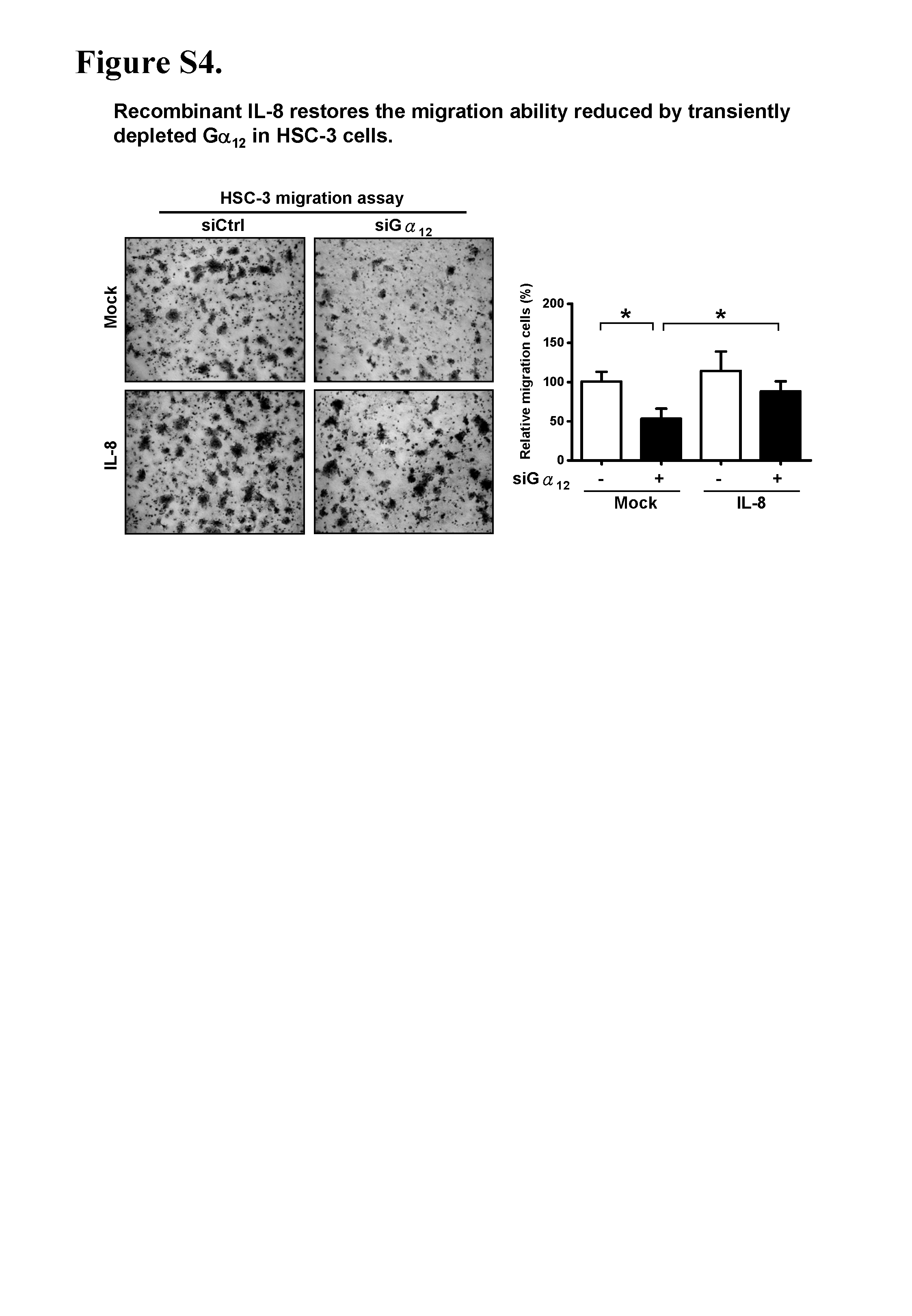

Supplement: Figure S4 — Recombinant IL-8 restores the migration ability reduced by transiently depleted Gα12 in HSC-3 cells. Representative images show the migration of the Gα12-depleted HSC-3 cells through transwells. Cells treated with or without recombinant human IL-8 (1 ng/ml) for 16 h. Quantification of migration is shown in the right panel. Error bars represent SD of the mean from three independent experiments and analyzed by t-test. *P<0.05. (TIF) [file pone.0066133.s005.tif]
